# The relationship between allergy and asthma control, quality of life, and emotional status in patients with asthma: a cross-sectional study

**DOI:** 10.1186/s13223-014-0067-4

**Published:** 2014-12-21

**Authors:** Hikmet Coban, Yusuf Aydemir

**Affiliations:** Department of Pulmonology, Sakarya University, Sakarya, Turkey; Training and Research Hospital, Sakarya University, 54100 Sakarya, Turkey

**Keywords:** Atopy, Asthma control, Quality of life, Emotional status, Depression, Anxiety

## Abstract

**Background:**

Psychiatric comorbidities are prevalent in patients with chronic somatic disorders such as asthma. But, there is no clear evidence regarding the effect of atopic status and the type of sensitized allergen on emotional status. The aim of the present study was to investigate the effects of house dust mites and pollen allergies on emotional status, asthma control and the quality of life in patients with atopic asthma.

**Methods:**

The study included 174 consecutive patients who were diagnosed with asthma accoring to the GINA criteria and who did not receive therapy for their allergy. All patients underwent a skin prick test. The asthma control, quality of life, and emotional status were evaluated using the ACT (asthma control test), AQLQ (asthma-specific quality of life questionnaire), and HAD (hospital anxiety depression questionnaire).

**Results:**

Atopy was detected in 134 (78.7%) patients. Of those patients: 58 (33.3%) had anxiety and 83 (47.7%) had depression. There was no relationship between emotional status, atopic status, and the type of indoor/outdoor allergen. Furthermore, there was no relationship between atopy and asthma severity, asthma control, and the quality of life. The anxiety and depression scores were significantly higher and the quality of life scores lower in the uncontrolled asthma group. The ACT and AQLQ scores were also lower in the anxiety and depression groups.

**Conclusions:**

It was concluded that anxiety and depression are prevalent in patients with uncontrolled asthma, and atopic status did not affect the scores in ACT, AQLQ, and emotional status tests.

## Introduction

Asthma is a chronic health problem that encompasses the patient’s entire lifetime, and causes significant mental and social problems in addition to physical symptoms. It is; therefore, considerably important to evaluate the quality of life of the patients in addition to the symptoms in order to gather full information about the health status of the patients [1].

Psychiatric comorbidities are frequently observed in patients suffering from chronic somatic disorders. These psychiatric comorbidities have a significant negative impact on the quality of life of the patients [1]. Emotional disorders, including anxiety and depression are more prevalent in asthma as compared to the general population [2-4]. Based on review of the literature, the prevalence of anxiety and psychological disorders among bronchial asthma patients can be estimated at 30–52% [5-7]. Similarly, high frequency of anxiety and depression has been demonstrated in atopic patients [8,9]. In that case, the association of asthma and the atopic trait further can contribute to the impairment in emotional status as compared to non-atopic patients with asthma. Also it can be a relationship between the type of allergen and emotional status. These last two topics have not yet sufficiently clear.

The allergens are well-known to cause asthma exacerbations and sustained symptoms [10]; however, there are still very few data regarding the effects of allergens on asthma control, quality of life, and emotional status. To our knowledge, there is no study that evaluated the relationship between the type of internal and external the allergens and emotional status.

The secondary aim of the present study was to assess the emotional status of patients with asthma. Main purpose of our study was to investigate the effects of house dust mites and pollen allergies on emotional status, asthma control and the quality of life in patients with atopic asthma.

## Materials and methods

The consecutive 174 patients, who were new diagnosed with asthma according to GINA criteria in Sakarya University Hospital (Turkey) in March-September 2012 period and who have not received at least last one month an antihistamines and immunotherapy for allergy and any inhaled or oral therapy for asthma were included in the present study. The patients who were receiving a therapy for psychiatric disease or those with a chronic disorder (chronic obstructive pulmonary disease, diabetes, coronary artery disease, malignancy) that could affect emotional status were excluded from the study.

A detailed medical history was obtained, and all patients underwent physical examinations. All pulmonary function tests were performed by the same technician on the SpiroAnalyser ST-300 instrument. The severity of asthma was determined based on Global Initiative for Asthma (GINA) criteria [1]. The patients, who provided informed consent, were included in the study, and the questionnaire form evaluating the demographic characteristics, anthropometric measurements, and the features of the asthma were administered to all patients. All patients filled out the ACT (asthma control test), AQLQ (asthma quality of life questionnaire) (Turkish version) and HAD (Hospital Anxiety and Depression) (Turkish version) surveys and the results were recorded on the questionnaire form. An approval was obtained from the university ethics committee.

### Assessment of asthma control

The patients were administered a 5-item questionnaire assessing their asthma symptoms, use of rescue medications, and the impact of asthma on daily life [11]. In asthma control test, a score of 25 points indicated full control, 20–24 points indicated controlled disease, 16–19 points indicated partial control, and score below 15 indicated uncontrolled disease. In statistical analysis, patients achieving a score higher than 20 were assessed as a single group (control and full control), and patients achieving a score lower than 19 were assessed as a separate group (partial control and uncontrolled). The validity and reliability of this questionnaire has been previously shown in adult Turkish patients with asthma [12].

### Assessment of the quality of life

The AQLQ assessed the scores of the patients regarding their symptoms (12 questions), restriction of activity (11 questions), emotional function (5 questions), and environmental exposure (4 questions). The total score was recorded as the average of the scores in 32 questions [13]. The answers to each question are scored one to seven (1 = maximum impairment and 7 = no impairment). Scoring domains were expressed as the mean score per question. Low scores indicate a poor quality of life.The validity and reliability of this questionnaire has been previously shown in adult Turkish patients with asthma [14].

### Assessment of emotional status

The assessment of emotional status was measured by using the HAD questionnaire. The Hospital Anxiety and Depression Scale was developed in 1983 by the Zigmond and Snaith and the validity and reliability were performed [15]. It includes the subscales of anxiety and depression. This scale consisted of a total of 14 questions: 7 of them investigated the symptoms of depression, and 7 of them investigated the symptoms of anxiety. The answers were evaluated in the form of a four-point Likert type scale. The scale was scored between 0 (best) and 3 (worst).

The purpose of the scale is not to diagnose, but to determine the risk group by scanning anxiety and depression in physically ill patients in a short amount of time. The scale can also be used in the follow up of changes in the patient’s emotional state. Although the scale uses the word “hospital”, it can also be used in the field or in a study performed in outpatient settings. To the aim was to minimize the effects of the existing somatic disease on the scale results in the HAD scale.

For this reason, it does not include any physical signs. In the preparation of scoring, “0 to 1” was recognized as normal, “2” was recognized as borderline symptomatic, and “3” was recognized as having prominent symptoms. It has been proven that the HAD scale is a useful assessment tool, and its score range provides results so as to minimize the false-positive and false-negative data [16]. It has been shown that scores obtained from the scale are not affected by the presence of somatic disease [16]. The HAD scale was used in comparison with other scales and was found to be adequate in terms of the evaluation of anxiety and depression in patients with somatic diseases [17].

Turkish adaptation study of HAD scala has been made by Aydemir et al. According to this study, for the anxiety sub-scale cut-off score has been found 10 and more, and depression subscale cut-off score has been found 7 and more [18]. Measurements displayed that the limit value for anxiety was 10 or higher, while the limit value for depression was 7 or higher. It was evaluated that an anxiety score greater than 10 was HAD-A (+), under 10 was HAD-A (−), and those having depression scores over 7 were HAD-D (+), while those under 7 were HAD-D (−).

### Evaluation with allergic skin test

A skin test (ALK-Abello, Madrid, Spain) was performed using 20 different aeroallergens in order to determine atopic status of the patients. The test media contained a mixture of Dermatofagoides farinea, D. pteronyssinus, storage mite, cockroaches, latex, hair, fungi, wheat, oak, olive, trees, mixture of weeds, cat hair, dog hair, grass mixture, and grass-wheat mixture. Histamine was used as a positive control and saline was used as a negative control. The test site was evaluated after 15 minutes, and the result was considered positive if the edema was more than 3 mm in diameter. The house dust mites were considered indoor allergen, and pollens were considered outdoor allergen (trees, mixture of weed, mixture of grass, grass-wheat mixture).

### Statistical analysis

The SPSS for Windows 21.0 (IBM) software package was used in the statistical analysis of the study. In the present study, the continuous variables were expressed with mean value, standard deviation, maximum and minimum values. The Shapiro-Wilk test was used for the normality test of continuous variables. Normally distributed continuous variables were compared by means of variables were evaluated with samples t test, nonparametric variables between the two groups were made by Mann–Whitney U test, categorical variables were compared by Chi square test.

## Results

### Sociodemographic features and general characteristics of asthma

A total of 174 patients were included in the study. The characteristics of the patients are presented in table 1.

**Table 1:** **Asthma and demographic characteristics of patients**

	**n**	**(%)**
Gender (F/M)	134/40	
Rhinitis	137	(78.7)
Atopy	137	(78.7)
	**Mean ± sd**	**Min/max**
Age	43.8 ± 13	18/72
BMI (kg/m^2^)	27.49 ± 5.2	17.70/49.30
FVC (%)	84.9 ± 16.5	23/129
FEV_1_ (%)	84.7 ± 19.3	19/125
FEV_1_/FVC (%)	84.4 ± 9.6	48/110
PEF (%)	67.4 ± 18.7	12/111
ACT	18.3 ± 5.1	5/25
AQLQ Total	4.3 ± 1.2	1.18/6.84

BMI: Body Mass Index; FVC: Forced Expiratory Volume; FEV_1_: Forced Expiratory Volume in 1.second; PEF: Peak Expiratory Volume; ACT: Asthma Control Test score; AQLQ; Asthma Quality of Life Questionnaire score.

### The prevalance of allergy and psychiatric disorders

The skin prick test was found to be positive for at least one allergen in 137 patients (78.7%), with 45 patients (25.9%) having only a pollen allergy, 32 patients (18.4%) having only a mite allergy, and 60 patients (34.5%) having both pollen and mite allergies. In total, 105 patients (60.3%) had pollen allergies and 92 patients (52.9%) had mite allergies.

The mean anxiety score was 8.07 + 4.42, the mean depression score was 7.00 + 4.54, and the mean HAD score was 15.07 + 7.97. When the cutoff for anxiety and depression was taken as 10 and 7, respectively, 58 patients (33.3%) had anxiety, and 83 patients (47.7%) had depression.

### ACT and AQLQ status

The mean ACT score was 18.32 + 5.1. Of the patients, 19 (10.9%) had fully controlled disease, 65 (32.4%) had controlled disease, 78 (44.8%) had partially controlled disease, and 12 (6.9%) had uncontrolled disease. When the cut-off for asthma control was taken as 20, 89 patients (51.1%) had uncontrolled asthma, and 85 patients (48.9%) had controlled asthma.

The mean total AQLQ score was 4.30 + 1.24, the mean symptom score was 4.76 + 1.38, the mean activity score was 4.04 + 1.27, the mean emotional score was 4.50 + 1.57, and the mean environmental exposure score was 3.61 + 1.64.

### The relationship between allergic status and ACT, AQLQ, and emotional status

There was no significant difference between patients who had versus who did not have mite and pollen allergy in terms of asthma control, quality of life, anxiety, and depression level (Table 2).

**Table 2:** **The relationship between atopy and emotional status, quality of life, and asthma control**

**Mean (SD)**	**Non-atopic asthma n = 37**	**Atopic asthma n = 137**	***p value***
HAD-Anxiety	8.43 (4.35)	7.98 (4.45)	*0.607*
HAD-Depression	7.57 (4.52)	6.85 (4.43)	*0.277*
HAD- Total	16.00 (7.51)	14.82 (8.10)	*0.302*
ACT	17.97 (4.89)	18.42 (5.18)	*0.486*
AQLQ-Total	4.45 (1.24)	4.29 (1.24)	*0.683*
AQLQ-Symptom	4.90 (1.34)	4.72 (1.39)	*0.550*
AQLQ-Activity	4.10 (1.35)	4.02 (1.25)	*0.855*
AQLQ-Emotional	4.63 (1.49)	4.47 (1.59)	*0.771*

HAD: Hospital Anxiety Depression Score; ACT: Asthma Control Test score; AQLQ; Asthma Quality of Life Questionnaire score.

Of the patients, 105 had pollen allergies, 92 had mite allergies, and 60 had both mite and pollen allergies. When analyzed by the type of allergens, there was no significant difference in terms of asthma control, quality of life, anxiety, and depression level (Table 3).

**Table 3:** **The relationship between the type of allergy and emotional status, quality of life, and asthma control**

**Mean (SD)**	**Pollen allergy**			**Mite allergy**		
	**Absent n = 69**	**Present n = 105**	***p value***	**Absent n = 82**	**Present n = 92**	***p value***
HAD-A	8.43 (4.42)	7.84 (4.42)	*0.327*	7.80 (4.27)	8.32 (4.56)	*0.444*
HAD-D	7.12 (4.31)	6.92 (4.56)	*0.561*	7.11 (4.28)	6.90 (4.62)	*0.486*
HAD-T	15.48 (7.83)	14.80 (8.07)	*0.418*	14.94 (7.59)	15.18 (8.33)	*0.947*
ACT	17.87 (5.34)	18.62 (4.95)	*0.392*	18.51 (4,98)	18.15 (5.24)	*0.757*
AQLQ-T	4.36 (1.32)	4.30 (1.19)	*0.675*	4.52 (1.20)	4.15 (1.26)	*0.099*

HAD-A: Hospital Anxiety Depression Scales Anxiety score; HAD-D: Hospital Anxiety Depression Scales Depression score; HAD-T: Hospital Anxiety Depression Scales total score. ACT: Asthma Control Test score; AQLQ; Asthma Quality of Life Questionnaire score.

### The relationship between emotional status and ACT and AQLQ

Independent from the type of allergen, the patients with an anxiety score greater than 10 and depression score greater than 7 had significantly worse asthma control and lower quality of life (Table 4).

**Table 4:** **The relationship between emotional status and the quality of life and asthma control**

**Mean (SD)**	**Anxiety**			**Depression**		
	**Absent n = 116**	**Present n = 58**	***p value***	**Absent n = 91**	**Present n = 83**	***p value***
ACT	19.28 (4.82)	16.41(5.17)	*<0.001*	20.18 (4.29)	16.20 (5.19)	*<0.001*
AQLQ-Total	4.68 (1.11)	3.62 (1.20)	*<0.001*	4.80 (1.07)	3.81 (1.21)	*<0.001*
AQLQ-Symptom	5.10 (1.21)	4.07 (1.46)	*<0.001*	5.27 (1.14)	4.20 (1.41)	*<0.001*
AQLQ-Activity	4.31 (1.24)	3.49 (1.15)	*<0.001*	4.44 (1.21)	3.59 (1.18)	*<0.001*
AQLQ-Emotion	5.08 (1.28)	3.36 (1.47)	*<0.001*	5.08 (1.33)	3.88 (1.58)	*<0.001*
AQLQ-Environ.	3.92 (1.63)	3.00 (1.48)	*<0.001*	4.03 (1.59)	3.16 (1.58)	*<0.001*
Asthma severity	1.91 (1.04)	2.21 (1.07)	*0.196*	1.81 (0.98)	2.22 (1.10)	*0.045*

ACT: Asthma Control Test; AQLQ; Asthma Quality of Life Questionnaire score. Environ: environmental.

### The relationship between allergic status and general patient characteristics

Atopic patients did not significantly differ in terms of spirometric measurements, BMI, age of asthma, and number of exacerbations; however, prick test positivity was significantly higher among male patients with rhinitis (Table 5).

**Table 5:** **The relationship between atopic trait and various patient characteristics**

**Mean (SD)**	**Non-atopic asthma n = 37**	**Atopic asthma n = 137**	***p value***
Age	46.73 (11.82)	43.06 (13.27)	*0.138*
Gender (f/m)	34/3	100/37	***0.016***
BMI (kg/m^2^)	28.20 (5.47)	27,30 (5.22)	*0.349*
Rhinitis (absent/present)	18/19	19/118	***<0.001***
FVC (% )	85.78 (15.59)	84.65 (16.81)	*0.551*
FEV_1_ (%)	85.14 (18.93)	84.79 (19.54)	*0.854*
PEF (%+)	67.46 (17.48)	67.3 (19.05)	*0.760*
Exacerbation/year	0.35 (0,6)	0.66 (1.61)	*0.868*
Asthma severity	2.05 (0,9)	1.99 (1,09)	*0.142*

BMI: Body Mass Index; FVC: Forced Expiratory Volume; FEV_1_: Forced Expiratory Volume in 1.second; PEF: Peak Expiratory Volume.

### The relationship between emotional status and general patient characteristics

The general characteristics and spirometric measurements were comparable between the two groups. The number of episodes was the only parameter that differed between patients with and without anxiety (p = 0.039), while the number of episodes (p = 0.002), and asthma severity (p = 0.013), were the two parameters that significantly differed between patients with and without depression.

### The relationship between asthma control status and emotional status

The patients with uncontrolled asthma achieved significantly worse scores in emotional status and quality of life domains (Table 6).

**Table 6:** **The relationship between ACT score and HAD-A and HAD-D**

**Mean (SD)**	**Uncontrolled asthma (ACT < 20) n:89**	**Controlled asthma (ACT ≥ 20) n:85**	***P value***
HAD-A	9.18 (4.40)	6.92 (4.16)	*0.001*
HAD-A ≥ 10 n (%)	37 (41,6)	21 (24.7)	*-*
HAD-D	8.27 (4.18)	5.67 (4.36)	*<0.001*
HAD-D ≥ 7 n (%)	54 (60.7)	29/85 (34.1)	*-*
HAD-T	17.42 (7.81)	12.61 (7.41)	*<0.001*
AQLQ	5.01 (1.13)	3.67 (0.95)	*<0.001*

HAD-A: Hospital Anxiety Depression Scales Anxiety score; HAD-D: Hospital Anxiety Depression Scales Depression score; HAD-T: Hospital Anxiety Depression Scales total score; AQLQ; Asthma Quality of Life Questionnaire score.

## Discussion

The present study evaluated the effects of the atopic trait and the type of allergy on the level of anxiety, depression, quality of life, and asthma control. When atopic patients with positive skin prick test were compared to non-atopic patients, no significant difference was found in terms of anxiety and depression scores, asthma control, and quality of life. There was also no significant difference in terms of the types of pollen and mite allergies.

The exposure to allergens is a well-established cause of asthma episodes and sustained symptoms in atopic patients [19,20]. In this case, atopic patients can be expected to have lower symptom scores and quality of life score, and hence higher anxiety-depression scores. This is due to the fact that anxiety and depression was shown to correlate with disease control [6,21-23]. However, the results provided evidences contrary to this hypothesis. As a matter of fact, whether atopy is associated with increased asthma control is a topic that has not been fully elucidated. In a study that evaluated atopic trait in asthma, no significant difference was found between atopic and non-atopic patients in terms of asthma symptoms, quality of life, hospitalizations, and admission to emergency room [24]. Both groups were found to be similar with regards to asthma severity [25]. In the present study, there was no significant difference between atopic and non-atopic patients in terms of asthma control, frequency of asthma episodes, asthma severity and spirometric measurements.

The major strength of the present study was its cross-sectional design and inclusion of patients with a recent diagnosis that have not received a previous allergic therapy. In studies that are based on the review of patient charts, treatment effect appears as a confounder, in that, therapy may have minimized the effects of allergy. Consequently, the studies that evaluated the effects of therapy have shown improvement in the quality of life score after the therapy in comparison to pre-treatment scores [26]. Another study reported a significant improvement in the quality of life of patients with house mite allergy that underwent immunotherapy [27].

There are a very limited number of studies in the literature regarding allergic sensitization and emotional status. Among these studies, Kohlbeck evaluated 182 atopic and 63 non-atopic children with asthma and reported a 3-fold higher rate of emotional disorders in the asthma group. Similarly to the present study, they did not find any significant differences between atopic asthma group and non-atopic group. In addition, they did not report a relationship between emotional disorders and age, gender, BMI, and frequency of rhinitis [28].

In the study by Barone et al. that evaluated 217 patients, the total Anxiety Sensitivity Index score was significantly higher in the atopic group when compared to the non-atopic group; however, no significant differences were found between atopic and non-atopic asthmatics in terms of their scores in Beck Depression Inventory-II. The spirometric measurements and severity of asthma were comparable between the two groups [29].

As a secondary outcome of the present study, ACT and ACLQ scores were found to be significantly lower in patients with anxiety and depression. Our study confirmed that both the prevalence and severity of emotional disorders are related to the degree of asthma control and quality of life. These results are consistent with the works of other authors. Janson observed a correlation between the prevalence of anxiety and depression and the occurrence of asthma symptoms. [30] In another study, anxiety and depression were more frequent among patients with “difficult” asthma [5]. In the study by Trzcińska et al., the prevalence of depression and its severity were significantly correlated with the degree of asthma control [6]. The results of Di Marco’s study show a significant correlation between poor level of asthma control and both anxiety (OR: 3.76) and depression (OR: 2.45), which are frequent disorders in asthmatic subjects [23]. The neuro-physiological mechanism of the relationship between depression and asthma has been evidenced in the previous studies [31,32].

Is the asthma control and quality of life worse in those with a psychiatric disorder, or may it be that emotional status is worse in patients with uncontrolled asthma? There is not a straightforward answer to this question as the present study has not been designed as to address this casual relationship. Age, BMI, presence of rhinitis, and as the basic parameters of asthma FEV_1_, FVC, and PEF values were similar in patients with anxiety and depression. However, the number of exacerbations were higher in the anxiety group, and the number of exacerbations and asthma severity were higher in the depression group. This discrepancy complicates the interpretation of the results. If these parameters were similar between the groups, this would provide convincing evidence that emotional status has negative effects on asthma control and the quality of life. In our opinion, anxiety and depression are more common in uncontrolled asthma group and the statistical significance of this relationship is stronger. Thus, it is more plausible to suggest that emotional disorders are the result of poor asthma control.

Of the patients in the study group, 78.7% had rhinitis. The prevalenece of house dust mite and pollen allergy was higher in patients with rhinitis. Only 18 patients (13%) had rhinitis combined with a negative prick test. Consistent with the current results, previous studies reported a rate of positive prick test in patients with allergic rhinitis that ranged from 81% to 62.5% [33-35]. No relationship was observed between rhinitis and emotional status.

Another major strength of the study is the use of objective skin prick tests to determine atopic status. And these tests were conducted at the same time with the asthma control, quality of life and psychological status evaluation.

Our study has its limitations; firstly, we used questionnaires to evaluate emotional status, and no actual physician diagnosis was used. Secondly, this study was a single center study, and it is not capable of evaluating various ethnic and sociocultural differences, thus preventing the generalized use of the study results.

The present study is the first, to our knowledge, to systematically investigate the association between emotional status, quality of life, and indoor-outdoor type of atopy in patients with asthma. Finally, it was concluded that house dust mite and pollen allergies did not influence the scores in ACT and AQLQ; therefore, they did not increase the prevalence of anxiety and depression. It was concluded that emotional impairment is prevalent in patients with uncontrolled asthma, and holistic treatment approach should include psychological support and patient education in addition to the provision of medical therapy.
